# Ultrasonographic/regional muscle measurements for diagnosing sarcopenia in older adults with and without dementia

**DOI:** 10.55730/1300-0144.5540

**Published:** 2022-06-26

**Authors:** Zekeriya ÜLGER, Gözde ŞENGÜL AYÇİÇEK, Özgür KARA, Murat KARA

**Affiliations:** 1 Department of Internal Medicine, Division of Geriatrics, Faculty of Medicine, Kırıkkale University, Kırıkkale, Turkey; 2 Department of Internal Medicine, Division of Geriatrics, Gülhane Faculty of Medicine, Ankara Oncology Education and Research Hospital, University of Health Sciences, Ankara, Turkey; 3 Department of Physical Medicine and Rehabilitation, Faculty of Medicine, Hacettepe University, Ankara, Turkey

**Keywords:** Alzheimer’s disease, gastrocnemius, grip strength, muscle thickness, ultrasound

## Abstract

**Background/aim:**

Sarcopenia and dementia are growing concerns among older adults that muscle and brain atrophy may cooccur. We aimed to compare the age-related loss of muscle mass by using ultrasound (US), and skeletal muscle mass index (SMI) by bioelectrical impedance analysis in older adults with and without dementia.

**Materials and methods:**

A total of 221 older adults aged ≥65 years were included in the study. The diagnosis of sarcopenia was established if low muscle mass according to either SMI or sonographic gastrocnemius (GC) muscle thickness was combined with low grip strength. The diagnosis of dementia was based on the National Institute of Aging and Alzheimer’s Association criteria and the major neurocognitive disorder definition in the Diagnostic and Statistical Manual of Mental Disorders-V. Muscle strength was measured by hand dynamometer and physical performance was assessed by 4-meter usual gait speed.

**Results:**

There were similar/moderate correlation coefficients between GC muscle thickness and SMI with functional parameters (all p < 0.01). Forty-six patients (20.8%) had dementia, and 21 (45.7%) of them had sarcopenia diagnosed by GC thickness (p < 0.001). Age was older but weight, body mass index, and all sarcopenia-related parameters were lower in dementia patients (all p < 0.01). When clinical variables were taken into binary logistic regression analyses, age [OR = 1.095 (95% CI: 1.028–1.167)], weight [OR = 0.918 (95% CI: 0.887–0.950)], and presence of dementia [OR = 5.109 (95% CI: 2.002–13.033)] were independently associated with sarcopenia diagnosed with GC muscle thickness (all p < 0.05).

**Conclusion:**

This study showed that sarcopenia is highly prevalent in older adults with dementia (45.7%) than without dementia (11.4%). Amongst different factors, increased age, having low body weight, and the presence of dementia independently increased the risk of sarcopenia diagnosed by GC muscle thickness (but not diagnosed by SMI) in older adults. Thus, we can evaluate easily and successfully the loss of (regional) muscle mass in dementia patients by using US in outpatient clinics.

## 1. Introduction

Sarcopenia is an age-related progressive loss of muscle mass and muscle function associated with adverse outcomes from the increased risk of functional decline to early death [1]. It can be defined as primary if it is due to only aging process or secondary if it is due to other causes such as comorbidities, malnutrition, or immobility. Among them, cognitive dysfunction is one of the most common cause of secondary sarcopenia by influencing the muscle function and physical performance [2]. In this regard, studies have indicated that sarcopenic patients are vulnerable to developing cognitive impairment, and sarcopenia is common among individuals with dementia [3,4].

Dementia is a neurodegenerative disease characterized by the deterioration of cognitive functions. The most common form of dementia is Alzheimer’s disease (AD), and its earliest sign is memory loss, and patients become dependent in daily life activities in the advanced stages [5]. With an aging population and improvements in life expectancy, the prevalence of dementia is expected to increase [6]. Because of the disease burden on health care expenditures, the researches on prevention and treatment of the disease are crucial.

Sarcopenia and dementia share common pathologies that as loss of lean mass has been associated with AD-related brain atrophy [7]. Presentation of sarcopenia in these individuals may be due to the reduction in physical activity levels that is both observed in those with dementia and linked with reductions in gray matter volumes [8].

As a component of sarcopenia diagnosis there are several methods to estimate muscle mass including anthropometric measurements, biochemical parameters, bioelectrical impedance analysis (BIA), and imaging tools such as dual-energy x-ray absorptiometry, computed tomography, magnetic resonance imaging, and ultrasound (US). Among imaging methods, the US seems promising and has superiority to others in terms of easy, portable, radiation-free, noninvasive, and repeatable properties. Its validity and reliability to assess muscle mass have been studied previously [9,10]. Accordingly, in this study, we aimed firstly, to compare the age-related loss of muscle mass by using US in older adults with and without dementia; secondly to compare sarcopenia diagnosed by gastrocnemius (GC) muscle thickness by US and skeletal muscle mass index (SMI) by BIA.

## 2. Materials and methods

Between October 2019–March 2020 a total of 530 older patients (≥65-year-old) from two geriatric out-patient clinics were included in this cross-sectional study. After the assessment of exclusion criteria, the study was concluded with 221 patients. Anthropometric measurements including weight, height, and waist and hip circumferences and chronic diseases were recorded. All patients underwent a comprehensive geriatric assessment. Patients with deteriorating general conditions, advanced dementia, pacemaker, prosthesis, severe edema, and electrolyte imbalance were excluded from the study. This study was approved by the local ethics committee (E-2021-38/23.06.2021) and it was performed in accordance with the ethical standards as laid down in the 1964 Declaration of Helsinki. All participants gave informed consent.

The diagnosis of dementia was based on the original National Institute of Aging and Alzheimer’s Association (NIA-AA) criteria for AD and the major neurocognitive disorder definition in the Diagnostic and Statistical Manual of Mental Disorders-V (DSM-V) [11,12].

The BIA measurement was taken using Bodystat Quadscan 4000 device (FL, USA) in the supine position. Electrodes were placed on the dorsal surface of the metacarpophalangeal and metatarsophalangeal joints, medially between the distal prominences of the radius and ulna and between the medial and lateral malleoli at the ankle. The SMI was calculated as described elsewhere [13].

The US evaluations were performed using a 5–12 MHz linear probe (Logiq 200 Pro, and Logiq P5 GE Medical Systems) by the same geriatricians (GŞA, ÖK). Measurements were performed unilaterally (dominant hand side) on the medial head of the GC muscle at the bulkiest area, in the prone position. Muscle thickness was determined as the distance between the upper and deeper aponeurosis with minimal compression.

Diagnosis of sarcopenia was based on definitions from the EWGSOP2 in 2018. The diagnosis of sarcopenia was established if low muscle mass (according to either SMI (<7.4 kg/m2 for females, <9.2 kg/m2 for males) [13] or sonographic GC muscle thickness (<13 mm for each sex) was combined with low grip strength (<19 kg for females, <32 kg for males) [14].

Muscle strength was measured by ‘Grip strength dynamometer, Grip D produced by Takei/Made in Japan with the dominant hand, and measurements were repeated for three times after 10-s intervals and the maximum handgrip strength value was recorded. Physical performance was assessed by a 4-meter usual gait speed. Walking speed ≤0.8 m/s was accepted as low physical performance.

### 2.1. Statistical analysis

Statistical analyses were performed using SPSS 21. Numerical variables are shown as mean **±** SD, categorical variables as N (%). Normal distribution was tested by Kolmogorov-Smirnov test (if sample size > 50) or Shapiro-Wilk test (sample size < 50). Continuous variables were compared by Mann Whitney U or Student’s t-test; while categorical variables were compared by chi-square or Fisher’s exact test, where appropriate. Correlations among demographic and sarcopenia-related parameters were assessed by Pearson or Spearman correlation coefficients in each sex. In addition, for predicting the presence of sarcopenia (either confirmed by SMI by BIA or GC muscle thickness by US), clinical variables were taken into binary logistic regression analysis (with backward likelihood ratio selection). Statistical significance was set at p < 0.05.

## 3. Results

The frequency of sarcopenia in older adults is shown in Figure. A total of 21 subjects (9.5%) had sarcopenia diagnosed by both SMI and GC muscle thickness, 10 subjects (4.5%) had sarcopenia diagnosed by SMI only, and 20 subjects (9.0%) had sarcopenia diagnosed by GC muscle thickness only.

A comparison of the clinical characteristics of older adults with and without dementia is shown in Table. 1. Among the participants, 46 of them (20.8%) had dementia. Patients with dementia were older than those without dementia subjects (p < 0.001). Weight, body mass index and all the sarcopenia-related parameters (i.e. GC muscle thickness, SMI, grip strength, and gait speed) were found to be lower in dementia patients than without (p < 0.01). The frequency of sarcopenia-diagnosed by GC muscle thickness-was higher in dementia patients (45.7% vs. 11.4%) than those without dementia subjects (p < 0.001).

Correlation analyses showed that there were similar/moderate correlation coefficients between skeletal muscle mass (SMM) and GC muscle thickness with grip strength and gait speed values (all p < 0.01) (Table 2). There were also only moderate correlations between sarcopenia diagnosed by GC muscle thickness and sarcopenia diagnosed by SMI (r = 0.625 for males, r = 0.464 for females, both p < 0.01).

Among 221 older adults, 31 (16 M, 15 F) subjects (14.0%) had sarcopenia confirmed by SMI, and 41 (13 M, 28 F) subjects (18.6%) had sarcopenia diagnosed by GC muscle thickness (Table 1). When clinical variables were taken into binary logistic regression analyses; age (OR = 1.152 [95% CI: 1.055–1.257], male sex [OR = 13.403 (95% CI: 3.211–55.952)] and weight [OR = 0.840 (95% CI: 0.788–0.894)] were independently associated with sarcopenia diagnosed by SMI (all p < 0.01) (Table 3). On the other hand, age [OR = 1.095 (95% CI: 1.028–1.167)], weight [OR = 0.918 (95% CI: 0.887–0.950)], and presence of dementia [OR = 5.109 (95% CI: 2.002–13,033)] were independently associated with the sarcopenia diagnosed with GC muscle thickness (all p < 0.01) (Table 4).

## 4. Discussion

This study showed that sarcopenia was more prevalent in older adults with dementia (in almost half) than without. Sarcopenia confirmed by GC muscle thickness or SMI had similar/moderate relations with functional parameters. Of note, amongst different factors, increased age, having low body weight, and the presence of dementia (about 5 times) are independently associated with the risk of sarcopenia diagnosed with GC muscle thickness (but not with SMI) in older adults.

In our study, we have found that dementia is independently associated with the development of sarcopenia. This relationship was prominent when binary logistic regression analyses were performed for sarcopenia diagnosed with GC muscle thickness. Loss of lean muscle mass in AD is found to be associated with brain atrophy and cognition [15]. Sarcopenia characteristics are often present in dementia patients. The link between these conditions may be either a consequence of AD pathophysiology or due to common mechanisms, such as inflammation [16,17]. Additionally, the presentation of sarcopenia in these individuals may be due to the reduction in physical activity levels or presence of malnutrition.

Normally, muscle thickness is positively correlated with weight and negatively correlated with height, that is, positively correlated with BMI [18]. Therefore, an increase in BMI in healthy adults leads to an increase in the muscle thickness measurements. As the normalized/adjusted cut-off values for GC muscle thickness are not available in the literature, the use of absolute values (<13 mm for each sex) may cause some errors. Therefore, having low weight values seemed to be a risk factor for sarcopenia. Additionally, since measurements of SMM by BIA were adjusted for height square, weak/slim subjects (such as nonobese male older adults) who had low grip strength might have been misdiagnosed as sarcopenic (overestimated), while those who are obese but having low grip strength (such as obese female older adults) might have been wrongly defined as normal (underestimated) [18]. As male older adults were taller and had lower body mass index than females and when SMM is adjusted with the height square males were misdiagnosed as sarcopenic. Indeed, in our study, as SMM was moderately to strongly related with weight and height in the correlation analysis, male older adults were found to be 13 times more at risk for sarcopenia diagnosed by SMI.

Of course, the interesting thing here is that although the majority of dementia patients (about 90%) had low muscle strength and slow walking speed (i.e. frail), dementia was not found as a risk factor for sarcopenia when diagnosed by SMI, however male sex was incorrectly determined as an important risk factor. In fact, this creates a disadvantage for the male sex (as having low muscle mass) since SMM was only adjusted by the height square. However, since sarcopenia affects muscles rich in type II (fast-twitch) fibers, measuring the affected muscles’ mass seems more rationale. In this regard, beside increased age and having low body weight, we found that presence of dementia independently increased (about 5 times) the risk of sarcopenia when diagnosed with GC muscle thickness in older adults.

We showed that, there were similar but moderate correlation coefficients between sarcopenia confirmed by GC muscle thickness and SMI with grip strength and gait speed values. It has been shown that GC muscle thickness measured by US predicts low muscle strength better than SMI measured by BIA [19]. There was a positive correlation between GC muscle thickness and handgrip strength in both sexes. Additionally, the authors mentioned that GC muscle thickness predicts sarcopenia and muscle strength in sarcopenic patients as well as SMI and other anthropometric measurements [19]. Recent years, the use of US in daily practice is rising and it has been shown that rather than loss of total muscle mass, regional muscle loss (i.e. volume, cross-sectional area, or thickness) can be more valuable in the diagnosis of sarcopenia [20]. As it has been mentioned previously, anterior thigh muscles undergo atrophy earlier with ageing, and this issue is paramount for the prevention of impairments and interventions [20]. Although we measured GC muscle thickness and used absolute values in this study, use of adjusted anterior thigh muscle thickness could give more accurate results [21].

The study has several limitations. First of all, it is a cross-sectional study and it is difficult to conclude that we could predict sarcopenia with US measures in dementia patients. While we can only talk about an independent association. Secondly, the small study population size limits the generalization of the interpretations.

In conclusion, this study showed that sarcopenia is highly prevalent (in almost half) in older adults with dementia. Of note, beside increased age and having low body weight, presence of dementia independently increased (about 5 times) the risk of sarcopenia diagnosed with GC muscle thickness (but not diagnosed with SMI) in older adults. Thus, we can evaluate easily and successfully the loss of (regional) muscle mass in chronic disorders such as dementia patients by using the US in outpatient clinics. Further studies in a longitudinal manner with larger populations are needed to investigate the relationship between cognitive dysfunction and sarcopenia.

## Figures and Tables

**Figure:**
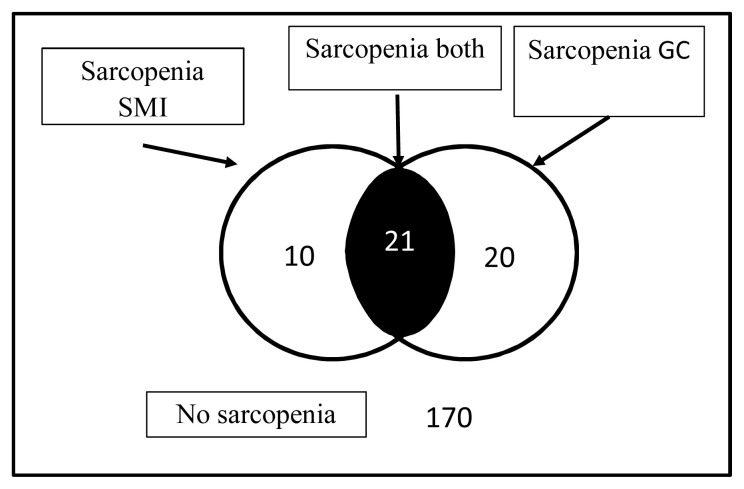
The frequency of sarcopenia in the older group (aged ≥65 years). Twenty-one individuals (9.5%) had sarcopenia according to both skeletal muscle mass index (SMI) and gastrocnemius (GC) muscle thickness, ten (4.5%) had sarcopenia diagnosed by SMI only, and twenty (9.0%) had sarcopenia diagnosed by GC muscle thickness only.

**Table 1:** Comparison of the clinical variables between older adults with/without dementia (N = 221).

Characteristic	Dementia (N = 46)	No dementia (N = 175)	p
**Age** (year)	79.0 ± 7.1	72.6 ± 6.3	**<0.001**
**Sex**, male	22 (47.8)	56 (32.0)	**0.046**
**Height** (cm)	157.6 ± 9.2	159.5 ± 9.4	0.157
**Weight** (kg)	68.8 ± 14.0	72.5 ± 13.8	**<0.001**
**BMI** (kg/m^2^)	27.8 ± 5.7	28.6 ± 5.8	**<0.001**
**Circumference** (cm)			
**Waist**	100.6 ± 16.1	99.6 ± 13.9	**<0.001**
**Hip**	104.4 ± 18.8	108.7 ± 11.0	**<0.001**
**Calf**	32.8 ± 4.4	35.6 ± 4.5	**<0.001**
**Comorbidities**			
**Hypertension**	38 (82.6)	113 (64.6)	**0.019**
**DM**	15 (32.6)	39 (22.3)	0.147
**CAD**	7 (15.2)	22 (12.6)	0.636
**Hyperlipidemia**	19 (41.3)	31 (17.7)	**0.001**
**CRD**	3 (6.5)	5 (2.9)	0.218
**Hypothyroidism**	5 (10.9)	17 (9.7)	0.500
**Grip strength** (kg)	18.5 ± 7.3	22.5 ± 8.7	**<0.001**
**Low grip strength**	39 (84.8)	86 (49.1)	**<0.001**
**Gait speed** (m/s)	0.49 ± 0.22	0.92 ± 0.37	**<0.001**
**Slow gait speed**	40 (87.0)	78 (44.6)	**<0.001**
**GC** (mm)	13.6 ± 3.2	15.8 ± 3.0	**<0.001**
**Low GC MT**	24 (52.2)	25 (14.3)	**<0.001**
**SMI** (kg/m^2^)	9.3 ± 2.0	9.9 ± 1.8	**<0.001**
**Low SMI**	13 (28.3)	21 (12.0)	**0.007**
**Sarcopenia by SMI**	13 (28.3)	18 (10.3)	**0.002**
**Sarcopenia by GC MT**	21 (45.7)	20 (11.4)	**<0.001**

Data are given as mean ± SD, or N (%).

Statistically significant variables are shown as bold.

N; number, BMI; body mass index, DM; diabetes mellitus, CAD; coronary artery disease, CRD; chronic renal disease, GC; gastrocnemius muscle thickness, SMI; skeletal muscle mass index, s; second.

* Statistical significance was caused by this value.

**Table 2:** Correlations among different sarcopenia diagnoses§ with grip strength and gait speed (N = 221).

	Data	SMM	GC MT	Age	Weight	Height	HGS	Gait speed
**Males** (N = 78)	**SMM**	-	0.545**	−0.257*	0.828**	0.511**	0.338**	0.381**
	**GC MT**	0.545**	-	−0.322*	0.478**	0.151	0.369**	0.420**
**Females** (N = 143)	**SMM**	-	0.336**	−0.349**	0.618**	0.588**	0.314**	0.401**
	**GC MT**	0.336**	-	−0.289**	0.263*	0.124	0.365**	0.250*

§ SMM; skeletal muscle mass, HGS; handgrip strength

GC MT; gastrocnemius muscle thickness

* p < 0.01,

** p < 0.001

**Table 3:** Binary logistic regression analysis for predicting sarcopenia diagnosed by SMI (N = 221).

Dependent variable	Independent variable*	Exp (β)	95% CI	P
Sarcopenia (N = 31)	Age	1.152	1.055–1.257	**0.002**
	Sex (male vs. female)	13.403	3.211–55.952	**<0.001**
	Weight	0.840	0.788–0.894	**<0.001**

β; standardized coefficients, CI; confidence interval

* Age, sex, weight, height, and presence of comorbidities are included in the analyses.

**Table 4:** Binary logistic regression analysis for predicting sarcopenia diagnosed by GC (N = 221).

Dependent variable	Independent variable*	Exp (β)	95% CI	P
Sarcopenia (N=41)	Age	1.095	1.028–1.167	**0.005**
	Weight	0.918	0.887–0.950	**<0.001**
	Dementia	5.109	2.002–13.033	**0.001**

β; standardized coefficients, CI; confidence interval

* Age, sex, weight, height, and presence of comorbidities are included in the analyses.
